# Wind Speed Prediction Based on Error Compensation

**DOI:** 10.3390/s23104905

**Published:** 2023-05-19

**Authors:** Xuguo Jiao, Daoyuan Zhang, Xin Wang, Yanbing Tian, Wenfeng Liu, Liping Xin

**Affiliations:** 1 School of Information and Control Engineering, Qingdao University of Technology, Qingdao 266520, China; jiaoxuguo@qut.edu.cn (X.J.); zdy431877849@gmail.com (D.Z.); tianyanbing@qut.edu.cn (Y.T.); xinliping_3968@126.com (L.X.); 2 State Key Laboratory of Industrial Control Technology, College of Control Science and Engineering, Zhejiang University, Hangzhou 310027, China; 3 Shandong Provincial Key Laboratory of Computer Networks, Shandong Computer Science Center, Qilu University of Technology, Shandong Academy of Sciences, Jinan 250014, China; 4 School of Civil Engineering, Qingdao University of Technology, Qingdao 266520, China; lwf6688@sohu.com

**Keywords:** Autoregressive Moving Average (ARMA), Support Vector Regression (SVR), Extreme Learning Machine (ELM), time series prediction, error compensation

## Abstract

Wind speed prediction is very important in the field of wind power generation technology. It is helpful for increasing the quantity and quality of generated wind power from wind farms. By using univariate wind speed time series, this paper proposes a hybrid wind speed prediction model based on Autoregressive Moving Average-Support Vector Regression (ARMA-SVR) and error compensation. First, to explore the balance between the computation cost and the sufficiency of the input features, the characteristics of ARMA are employed to determine the number of historical wind speeds for the prediction model. According to the selected number of input features, the original data are divided into multiple groups that can be used to train the SVR-based wind speed prediction model. Furthermore, in order to compensate for the time lag introduced by the frequent and sharp fluctuations in natural wind speed, a novel Extreme Learning Machine (ELM)-based error correction technique is developed to decrease the deviations between the predicted wind speed and its real values. By this means, more accurate wind speed prediction results can be obtained. Finally, verification studies are conducted by using real data collected from actual wind farms. Comparison results demonstrate that the proposed method can achieve better prediction results than traditional approaches.

## 1. Introduction

Since the beginning of the 21st century, people’s demand for energy has become stronger and stronger [1]. However, coal, oil, natural gas and other non-renewable resources still occupy the main part of the world energy market [2]. The burning of these fossil fuels will release greenhouse gases and threaten the environment. In order to solve the energy crisis and protect the environment, people are trying to develop new energy sources [3]. Nowadays, as one of the fastest developing new technologies in recent years, wind power generation technology is attracting widespread attention [4].

Wind power is a clean, renewable and pollution-free energy source [5]. However, as a natural resource, wind power has the characteristics of instability and uncertainty. Wind speed changes frequently, which brings great challenges to the stability of the wind power generation system and increases the operation and maintenance costs of the power plant [6].

In order to solve the instability problem in wind power generation, one of the most effective solutions is wind speed prediction [7]. As a cheap and effective method, it also plays a positive role in reducing operating costs and improving wind power competitiveness [8]. Through predicting the wind speed, it can guide the power scheme to adjust the power generation plan reasonably [9], change the torque of a wind turbine to maximize the use of wind power [10], protect the safety of the wind turbines so that the wind farm can use more advanced materials [11,12,13], and optimize the layout of wind turbines and improve the economic benefits of wind farms [14].

Many methods have been proposed for wind speed prediction. These methods can be divided into two categories: linear strategies and nonlinear approaches. Traditional linear methods use the linear combination of historical values to predict future wind speeds, such as with Autoregressive Moving Average (ARMA) [15]. Since these methods assume the relationship between historical wind speeds and future ones are linear and ignore the nonlinear characteristics, the prediction accuracy is not optimal [16]. With the rise of artificial intelligence [17], more and more nonlinear methods have been studied, such as Backpropagation Neural Network (BPNN) [18], Convolution Neural Network (CNN) [19], Support Vector Machine (SVM) [20], Extreme Learning Machine (ELM) [21], Long-short Term Memory Network (LSTM) [22], and the hybrid model combining multiple methods [23]. These nonlinear methods can accept more input features and can achieve more accurate predictions by selecting appropriate activation functions and hyperparameters.

However, to reduce the operating costs, some wind farms only collect and save wind speed information for wind speed forcasting. They ignore other factors affecting wind speed, such as topographic features, temperature, pressure, humidity and so on [24]. This causes the original data to be a univariate time series containing historical wind speeds only. Many nonlinear methods, such as ANN, SVR, ELM, etc., need sufficient inputs to ensure the accuracy of the prediction results. For univariate time series, if we want to achieve better prediction accuracy by using machine learning methods with a relatively low computation cost, we may need to divide the sequence into multiple segments and choose the appropriate number of data as the input and output for constructing a training set. That is, an appropriate number of historical wind speed points should be used to predict future values. Moreover, in the forcast methods based on time series, there is always a time lag between the predicted wind speed and actual values due to the rapid changes in natural wind speeds and time delays in the original wind data collection. This prediction lag will greatly reduce the accuracy of the wind speed forecast results [25].

Therefore, to find the optimal number of historical input data for a prediction model and alleviate the forecast time lag phenomenon, this study proposes a novel wind speed prediciton method to improve the prediction accuracy by combining the ARMA-SVR model and an error compensation technique. First, the ARMA model of the raw wind speed data is built, and the Partial Autocorrelation Coefficient (PAC) *p* of the model is calculated by using Akaike Information Criterion (AIC). Second, the *p* value is used as the basis for dividing the data set to train SVR for the wind speed prediction model. Then, the error set can be constructed by subtracting the predicted value from the true value. The same data preprocessing method for raw wind speed is utilized for the error time series to obtain the training set for the lag compensation model. Finally, the ELM is employed as the time lag correction model to give the predicted error. By adding the raw wind speed prediction result and the error prediction result, the final wind speed prediction result can be obtained. Through a comparison with SVR and BPNN, the ARMA-SVR-ELM model in this paper has a better prediction effect. The major contributions of this paper can be summarized as follows:

- For univariate time series forecasting, in order to explore the balance between the computation cost and the sufficiency of input features, this paper uses the parameters of the ARMA model which can be employed as the basis from which to select the optimal division data as the input of the SVR model.
- Through constructing the error dataset, an ELM-based time lag compensation technique is designed to mine the effective information of the error time series. By this way, the forecast lag phenomenon can be alleviated effectively.
- By combining the ARMA-SVR model and the error correction approach, a novel wind speed prediction approach is proposed to improve the prediction results. Simulation results show the proposed method can achieve better accuracy than traditional methods.

The rest of this paper is arranged as follows. Section 2 introduces the mathematical models of ARMA, SVR and ELM. Section 3 introduces the proposed method. Section 4 introduces the experiment and discusses the results. Section 5 draws conclusions.

## 2. Mathematical Models

This paper mainly uses ARMA, SVR and ELM models, which will be briefly described in this section.

### 2.1. The ARMA Model

The ARMA (*p*, *q*) model is one of the earliest models used for time series prediction [26]. It includes two parts: the Autoregressive(AR) model and the Moving Average(MA) model. The ARMA model can be described as follows

(1) $$X_{t}=\sum_{i=1}^{p}\alpha_{i}X_{t-i}+\sum_{j=1}^{q}\beta_{j}\epsilon_{t-j}+\epsilon_{t},$$

where $X=\left\{X_{1},X_{2},\ldots ,X_{t}\right\}$ is wind speed time series.

The ARMA model describes the relationship between current value, historical error and historical value. Equation (1) shows that the current value $X_{t}$ is composed of a linear combination of *p* historical values and *q* historical errors. The *p* and *q* are selected by AIC. The *p* value means that if every *p* data is taken as a group, the internal correlation of this group is very strong. Therefore, it is very reasonable to select *p* as the basis for dividing the univariate series.

### 2.2. The SVR Model

The SVR model can map low dimensional nonlinear problems to high dimensional space [27], and transform them into linear problems. The optimal hyperplane can be found as follows

(2) $$f\left(x\right)=\omega^{T}\Phi \left(x\right)+b.$$

In this paper, the Radial Basis Function (RBF) is chosen as the kernel function. The RBF can map samples to a higher dimensional space, which can better handle nonlinear wind speed prediction problems. The RBF is shown as follows

(3) $$k\left(x,x^{\prime }\right)=e^{-\frac{||x-x^{\prime }{\left|\right|}^{2}}{2\sigma^{2}}},$$

where $||x-x^{\prime }{\left|\right|}^{2}$ is the Euclidean distance (L2 norm) of *x* and $x^{\prime }$, and σ controls the range of influence of the RBF. The larger the σ is, the larger the influence range of the RBF.

To find the the optimal hyperplane f(x), hyper parameter ω and *b* need to be optimized. Therefore, the SVR model minimizes the following constrained condition

(4) $$\min_{\omega ,b,\xi ,\xi^{*}}\frac{1}{2}{∥\omega ∥}^{2}+C\sum_{i=1}^{N}\left(\xi +\xi^{*}\right)$$

(5) $$s.t.\left\{\begin{array}{c} y_{i}-f\left(x_{i}\right)\leq \varepsilon +\xi^{*}\\ f\left(x_{i}\right)-y_{i}\leq \varepsilon +\xi \\ \xi ,\xi^{*}\geq 0. \end{array}\right.$$

The SVR model can achieve a good prediction effect due to its ability to describe the nonlinear relationship between an input and an output [28]. Therefore, this paper chooses the SVR model to predict future wind speed.

### 2.3. The ELM Model

The ELM is a single hidden layer network model that uses random input layer weights and deviations and a generalized inverse matrix theory to calculate the output layer weights. Its output $y_{i}$ is as follows

(6) $$y_{i}=\sum_{j=1}^{K}\lambda_{j}\cdot g\left(\omega_{j}\cdot x_{i}+b_{j}\right),i=1,2,\ldots ,N.$$

ELM is not sensitive to the selection of parameters, and it has a fast training response and high accuracy [29]. Therefore, this paper uses the ELM model to predict errors.

### 2.4. Model Evaluation Index

This paper uses Root Mean Square Error (RMSE) and Coefficient of Determination ($R^{2}$) to evaluate the prediction effect of the model

(7) $$RMSE=\sqrt{\frac{1}{N}\sum_{i=1}^{N}{\left({\hat{x}}_{i}-x_{i}\right)}^{2}}$$

(8) $$R^{2}=1-\frac{\sum_{i=1}^{N}{\left(x_{i}-{\hat{x}}_{i}\right)}^{2}}{\sum_{i=1}^{N}{\left(x_{i}-{\bar{x}}_{i}\right)}^{2}}.$$

From Equations (7) and (8), *N* is the number of samples, $x_{i}$ and ${\hat{x}}_{i}$ represent the ith real value and the ith estimated value of the wind speed series, respectively, and ${\bar{x}}_{i}$ is the average value of the samples.

## 3. The Proposed Method

In order to better predict univariate time series, this paper proposes an ARMA-SVR wind speed prediction method based on ELM error compensation (ARMA-SVR-ELM hybrid model).

The data used in this paper are the univariate series, which means the data only has wind speed without other features. In order to enable SVR and ELM models to make full use of the original data with appropriate computational cost, this paper uses the ARMA model to find the relationship between each data point in the original univariate sequence, and it takes several data with strong correlation as a new set of sequences. As described in Section 2, the ARMA model can effectively handle the time univariate series. It can be seen from Equation (1) that in the ARMA model, the current value is related to the partial historical value and the historical error value. These values have a relatively strong correlation. Therefore, it is reasonable to use each group of sequences that has strong correlation as the training parameters of SVR to predict future wind speed. In this new set of sequences, the last value is used as the output, and all the previous data are used as the inputs. By this means, the original data is divided into multiple groups, and all of them are used to train the SVR and ELM models. Since the ARMA model is often used to deal with univariate time series, the ARMA model’s PAC reflects the relationship between the current value and multiple historical values with strong correlation. Therefore, it is very reasonable to divide the original data into multiple groups of sequences based on the *p* value obtained by the ARMA method.

This paper uses the divided data to train the SVR model to predict wind speed directly. Although the overall effect looks good, the fitting effect is not ideal in some periods when the wind speed changes rapidly. In order to deal with this and further improve the wind speed prediction accuracy, the prediction error was collected to develop the error correction technique in our study. The collected error data is still a univariate time series. Therefore, the same ARMA processing method as that of the original wind speed data was adopted, and the divided data was used to train the ELM model for error prediction. By this means, the error value of the future wind speed was obtained. When getting the wind speed prediction value and the error prediction value, the final wind speed prediction value can be obtained by adding these two prediction values. This process is called the “wind speed prediction based on error compensation technique” in our study.

**Figure 1:**
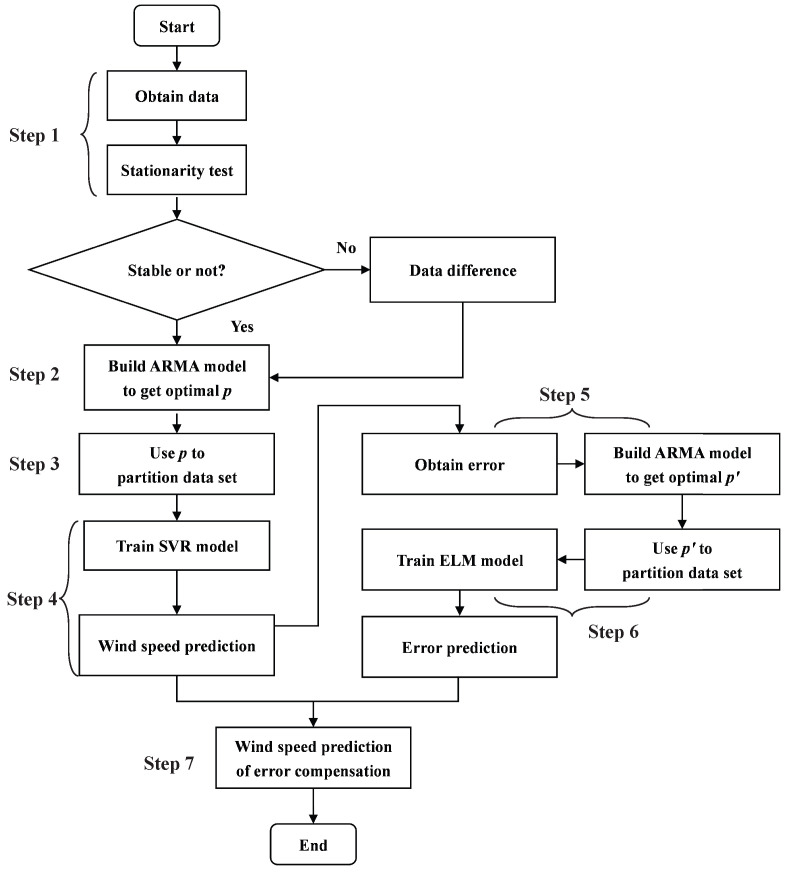
The Flow Chart of The Experiment.

The flow chart of the proposed method is shown as Figure 1 and the detailed description is as follows.

- Step 1: Data Processing

The ARMA model is often used for time series prediction, and it requires that the input data must be a stationary series. The definition of a stationary sequence is as follows

(9) $$\left\{\begin{array}{c} E\left(Y_{t}\right)=\mu \\ \mathrm{Var}\left(Y_{t}\right)=\sigma^{2},\\ \gamma_{t,t-k}=\gamma_{0,k} \end{array}\right.$$

where $\gamma_{t,t-k}$ represents the autocorrelation coefficients at time *t* and time t−k.

Equation (9) shows that the expectation and variance of the stationary sequence do not change with time. Due to the influence of temperature, pressure and many other factors, there are few stable wind speed time series in nature. This leads to the fact that the data collected in many cases cannot be used directly and must be preprocessed. In order to make the sequence stable, this paper uses differential processing, and the Augmented Dickey-Fuller (ADF) test is used to check whether the differenced sequence is stable. After that, the Autocorrelation Functions (ACF) and the Partial Autocorrelation Functions (PACF) diagrams are calculated to determine the value range of the number of input features.

In Step 1, the ADF test is used to test the stationarity of the sequence by checking whether the characteristic root of the sequence is within the unit circle.

- Step 2: ARMA Modeling

This step uses the differential data to establish the ARMA (*p*, *q*) model, and then AIC criteria are used to find the optimal *p* and *q*. In the above-mentioned model, *p* represents Partical Autocorrelation Coefficient (PAC) and *q* represents Autocorrelation Coefficient (AC).

In Step 2, AIC is often used to evaluate the quality of the regression model. In this paper, it is used for determining the optimal AC and PAC of the ARMA(*p*, *q*) model. The AIC can be described as follows

(10) $$AIC=nln(\frac{SSR}{n})+2k,$$

where SSR is Sum of Squared Residuals, *n* is the number of samples, and *k* is the number of unknown parameters.

- Step 3: Data Partitioning

After finding the optimal *p*, we use it to partition the data. With this specific method, starting from the data at the first moment, each *p* data is a group, where the first p−1 data are the inputs, and the *p*th data is the output. That is, the first p−1 data are used as the features to predict the *p*th value. Then, we start with the second data set and repeat the previous step until all the data are divided. This process can be described as follows

(11) $$\begin{array}{c} {\hat{x}}_{p}=f_{p}\left(x_{1},x_{2},\ldots ,x_{p-1}\right)\\ {\hat{x}}_{p+1}=f_{p}\left(x_{2},x_{3},\ldots ,x_{p}\right)\\ \vdots \\ {\hat{x}}_{\mathrm{end}\;}=f_{p}\left(x_{\mathrm{end}\;-p+1},x_{\mathrm{end}\;-p},\ldots ,x_{\mathrm{end}\;-1}\right). \end{array}$$

The principle for this is that *p* represents the correlation of the data in the original sequence, which can maximize the utilization of the data without losing too much machine performance.

- Step 4: Wind Speed Prediction

The partitioned data are used to train the SVR model to predict the wind speed. In this step, the training data and the test data are from Step 3, and the choice of kernel function for the SVR model is RBF.

- Step 5: Error Data Processing and Modeling

The trained SVR model obtained in Step 4 is used to predict the training set, and then the predicted value of the original data can be obtained. The error sequence can be obtained by subtracting the predicted value from the original data. The processing and modeling method of the error sequence is the same as that of the raw data in Step 1 and Step 2. The stationarity of the sequence is detected first. Then, its ACF and PACF images are drawn, and the ARMA model is built to find the optimal *p* and *q* of the error set by using the AIC criteria.

- Step 6: Error Data Partitioning and Predicting

The method of dividing error data is the same as in Step 3: the data are divided into multiple groups by using the *p* value obtained in Step 5. The first p−1 numbers of each group are regarded as the inputs, and the *p*th data is regarded as the output. Then, the partitioned data are used to train the ELM model to predict the future error.

- Step 7: Get the Final Wind Speed Prediction Result

In Step 4, we get the wind speed prediction result, and the error prediction result is obtained in Step 6. In this step, we need to add them, and then the final wind speed prediction result can be obtained.

## 4. Experiment

**Figure 2:**
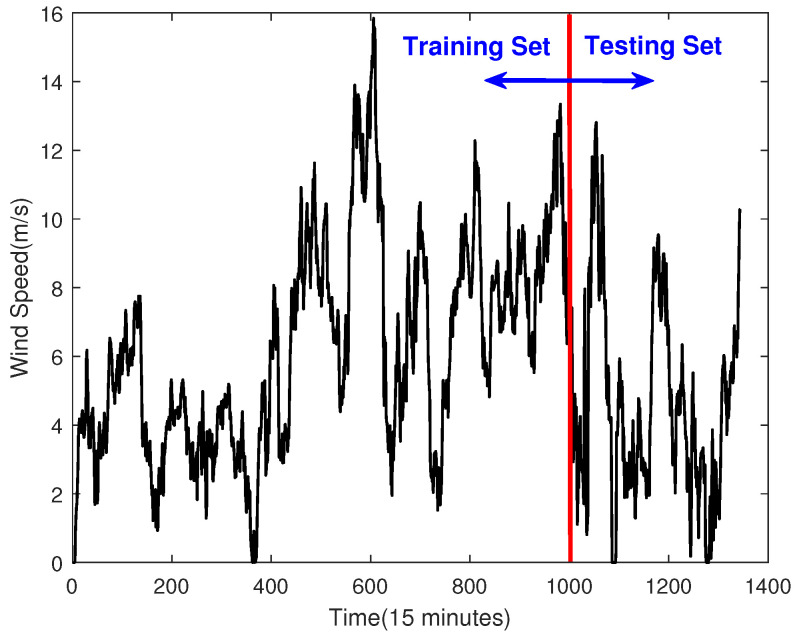
The Raw Data.

This section mainly introduces the results and discussions of the experiment. The raw wind speed data from a wind farm in China is shown in Figure 2. Among them, the data are recorded every 15 min, a total of 1344 data points are selected, the first 1000 data points are used as the training set, and the remaining data are used as the testing set. The experiment was tested on a PC with AMD Ryzen 5800H, NVDIA RTX 3070 Laptop and 16 GB memory. The following is the experimental result.

### 4.1. Experiment Steps of Wind Speed Prediction

#### 4.1.1. Data Processing

**Figure 3:**
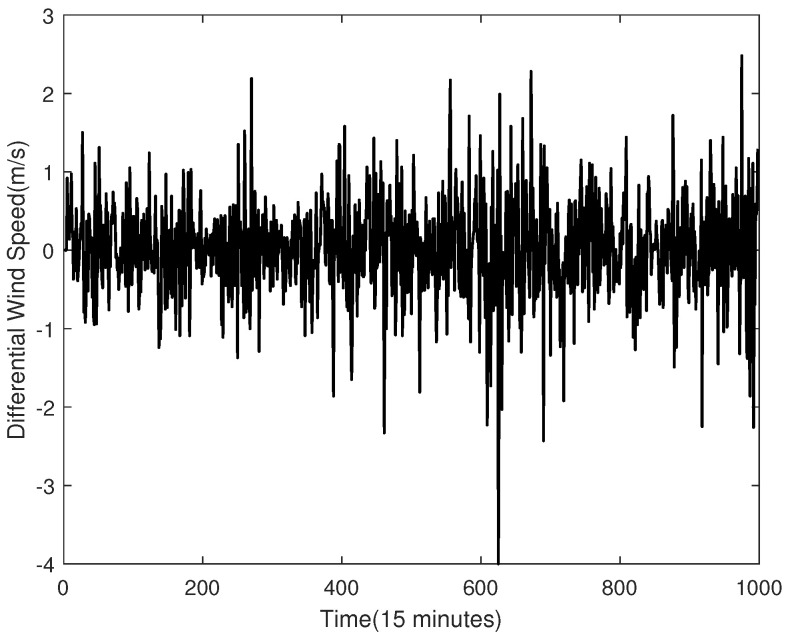
The Raw Data After Difference.

**Figure 4:**
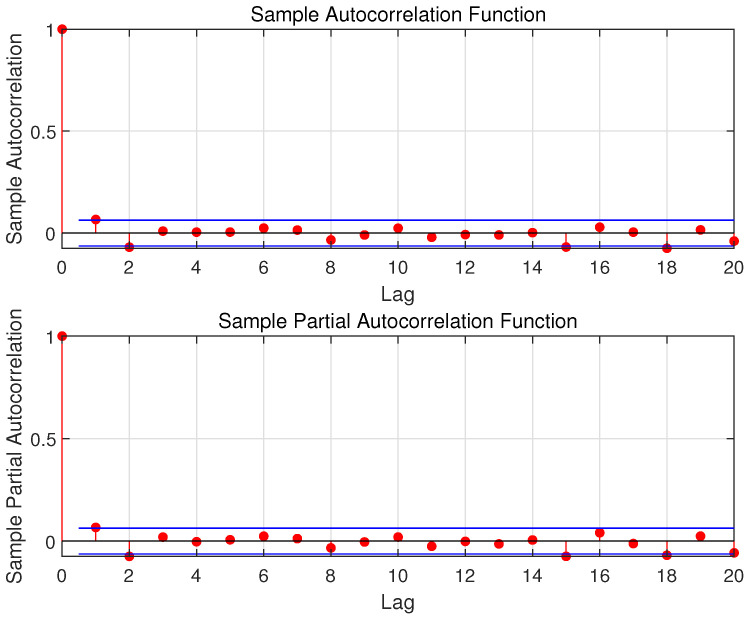
The ACF and PACF of Raw Data (The blue lines are the upper and lower confidence bounds).

By using the ADF test operation, we prove that the raw data shown in Figure 2 are not a stable sequence. In order to make the sequence stable, this paper uses differential processing. It is worth noting that the testing set is considered unknown, and therefore, the training set is used to do the differential processing. The processing result is shown in Figure 3, and its ACF and PACF are shown in Figure 4. In this paper, the raw data after using the differential operation are used to obtain the ARMA model.

The differential wind speed is shown in Figure 3. The ADF test is also used in this case, and the result also proves that the differential wind speed is a stationary sequence. Thus, it can be used for ARMA modelling.

#### 4.1.2. ARMA Modeling

This step uses the differential data to establish the ARMA (*p*, *q*) model. The selection of *p* and *q* is an important step in the ARMA model. Figure 4 shows the ACF and PACF of the original data. Note that as ACF and PACF decrease, the relationship between historical wind speed and the future one becomes weaker and weaker. To guarantee the balance between the computation cost and the sufficiency of input features, the value range of *p* and *q* are respectively chosen as 3 to 7 and 4 to 7 [30]. Then, the AIC is used to find the relatively appropriate *p* and *q* in the ARMA (*p*, *q*) model. The specific method is to set a cycle and use the iterative method for each combination of *p* and *q*. The relatively appropriate values of *p* and *q* can be obtained when the AIC value is the minimum.

As shown in Table 1, the minimum AIC value is obtained when p=4 and q=5. This means that the optimal value in data portioning should be 4.

#### 4.1.3. Data Partitioning

The optimal *p* is used to partition data. Every four data are divided into a group. The first three data are regarded as inputs, and the fourth data point is regarded as a target. The same processing is employed to all the wind speed data until all of them are portioned. This process is described by the following equation

(12) $$\begin{array}{c} x_{4}=f_{p}\left(x_{1},x_{2},x_{3}\right)\\ x_{5}=f_{p}\left(x_{2},x_{3},x_{4}\right)\\ \vdots \\ x_{\mathrm{end}}=f_{p}\left(x_{\mathrm{end}-3},x_{\mathrm{end}-2},x_{\mathrm{end}-1}\right). \end{array}$$

#### 4.1.4. Wind Speed Prediction

**Figure 5:**
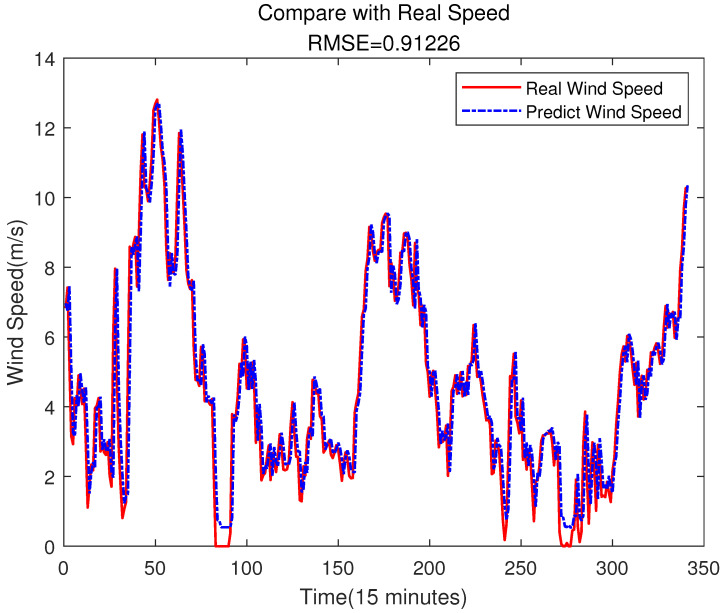
Wind Speed Prediction of SVR.

The SVR model can be trained by the partitioned data in Section 4.1.3, and then we can use the trained model to predict the wind speed. In this experiment, the choice of kernel function of the SVR model is RBF. The parameter of RBF σ is 0.0039, and the value of the penalty factor *C* is 6.285409. Both of them are selected by the trial and error method. The predicting result is shown in Figure 5.

**Figure 6:**
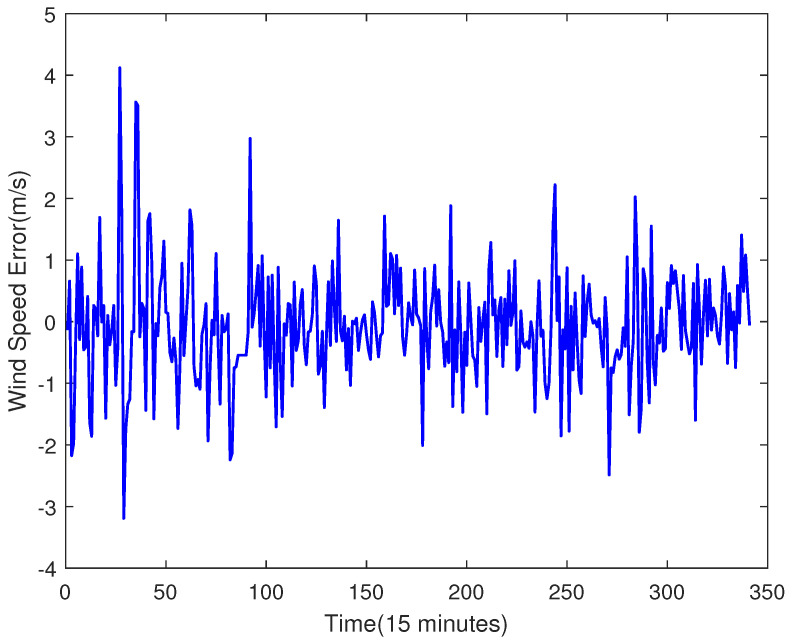
The Prediction Error.

It can be seen from Figure 5 that the SVR model can effectively predict the change in wind speed on the whole. The prediction error is shown in Figure 6.

### 4.2. Experiment Steps of Error Prediction

In order to further improve the prediction accuracy, an error compensation prediction based on ELM is used in this paper. In this method, the ELM is used to predict the error. Then, a more accurate wind speed prediction result can be obtained by adding the the error prediction result and the wind speed prediction result.

#### 4.2.1. Error Data Processing and Modeling

**Figure 7:**
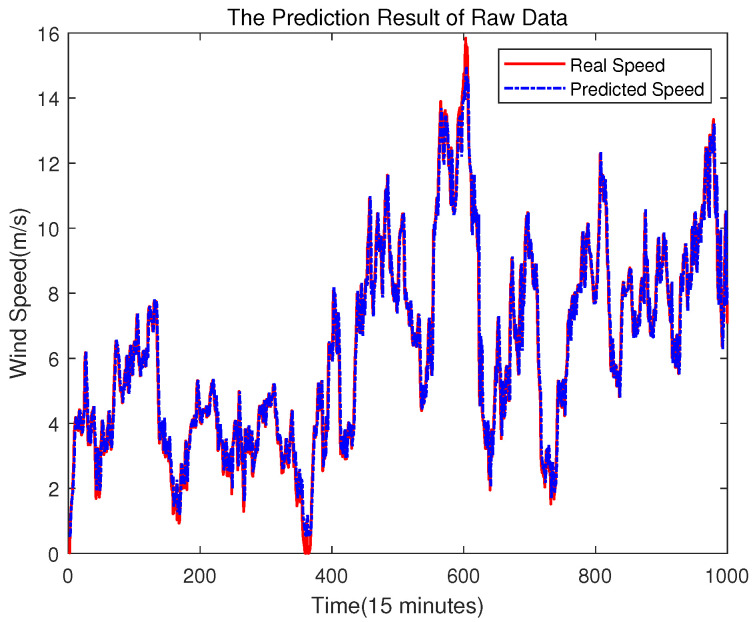
The Result of Raw Data Prediction.

**Figure 8:**
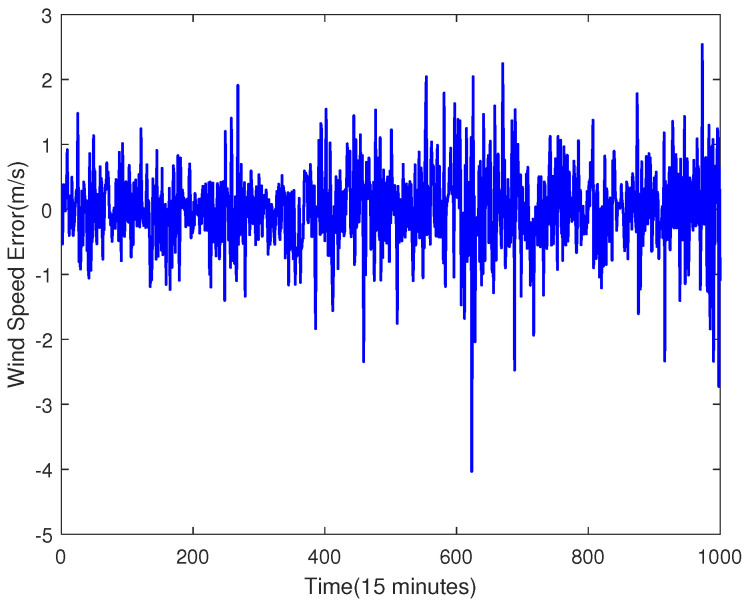
The Raw Error Data.

The trained SVR model is used to predict the original data, which is shown in Figure 7. Then, the error sequence can be obtained by subtracting the predicted value from the original data, which is shown in Figure 8.

Like the original data, the error data are also a univariate time series. Therefore, the data processing method is the same as that of the original data.

**Figure 9:**
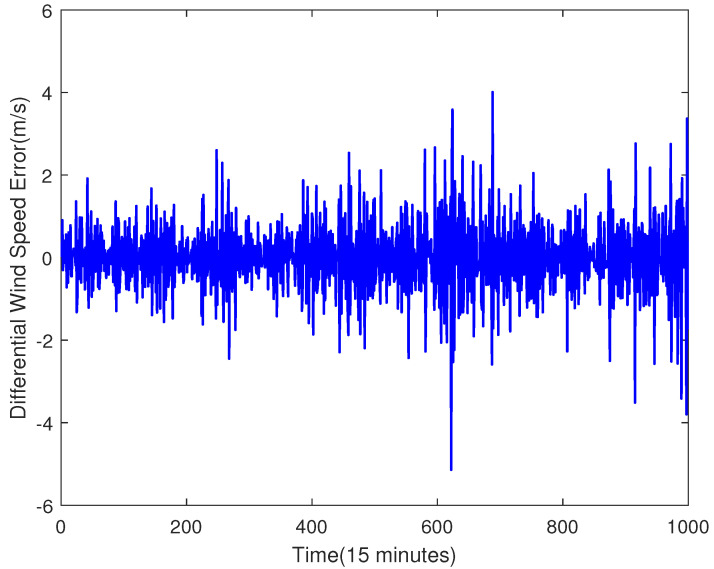
The Differential Error Data.

**Figure 10:**
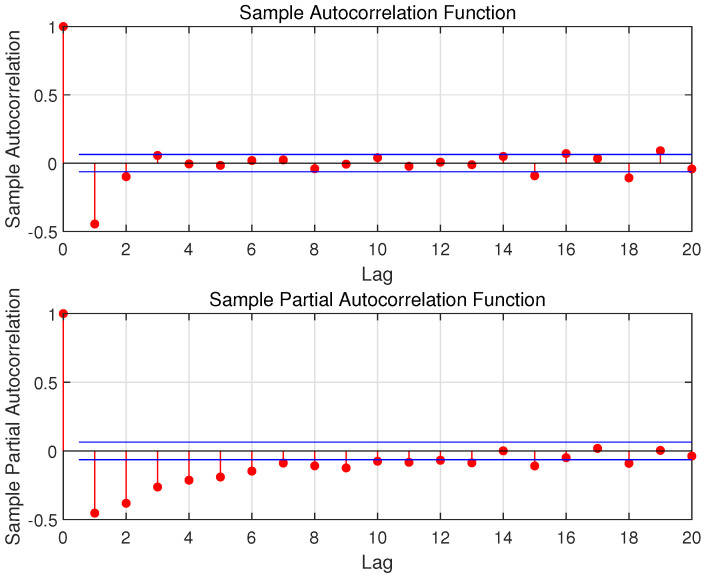
The ACF and PACF of Error Series (The blue lines are the upper and lower confidence bounds).

As shown in Figure 8, the sequence looks stable, but it does not pass the ADF test. Therefore, the sequence needs to be differentiated. The differential result is shown in Figure 9, and the ACF and PACF are shown in Figure 10.

It can be estimated from Figure 10 that the optimal values of *p* and *q* in ARMA (*p*, *q*) should be both around 5 to 7. Therefore, the AIC criterion is used to find the most reasonable values of *p* and *q*. The result of AIC is shown in Table 2.

As Table 2 shows, the minimum AIC value is obtained when p=5, q=3. Therefore, the theoretical optimal ARMA model is ARMA(5, 3).

#### 4.2.2. Error Data Partitioning and Predicting

**Figure 11:**
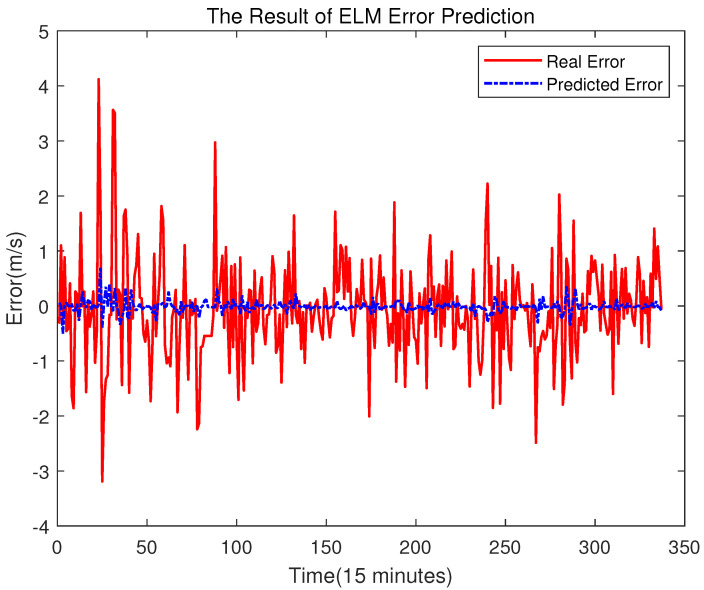
Error Prediction of ELM.

The method of dividing data is the same as before: the *p* value is used to divide the data into multiple groups. The first p−1 numbers of each group are regarded as the input, and the *p*th data is regarded as the target. Then, the partitioned data are used to train the ELM model to predict the future error. In this step, the activation function of the ELM model is the Sigmoid Function, and the number of hidden layer neurons is 10. The result of the error prediction is shown in Figure 11.

From Figure 11, one may see that the deviations between the real error and the predicted one is relatively big. However, for error compensation, as long as the changing trend is predicted correctly, the accuracy of the final predicted result will also be improved. As shown in Figure 11, the changing trend of wind speed error is accurately predicted by using the proposed error correction technique. Thus, more accurate wind prediction results can be achieved after error compensation in our study.

#### 4.2.3. Add Error Prediction and Wind Speed Prediction

After the results of wind speed prediction and error prediction are obtained, the final wind speed prediction result can be obtained by adding them up.

**Figure 12:**
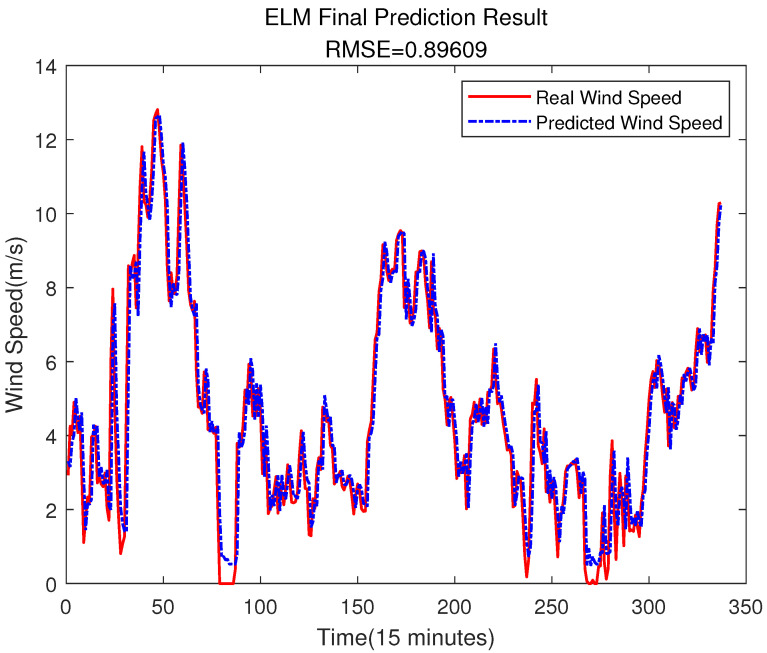
The Final Prediction Result.

It is worth noting that due to the use of error compensation, the first few values in Figure 5 will be used as inputs to predict future values, which results in different images in Figure 5 and Figure 12 at the beginning, but this does not affect the experimental results.

**Figure 13:**
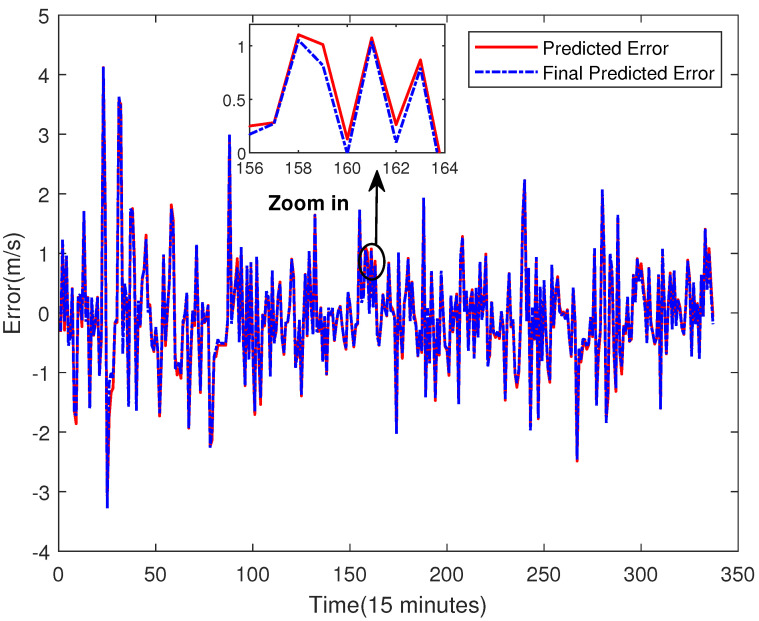
The Error Comparison.

Compared with the direct prediction of wind speed (shown as Figure 5), Figure 12 shows that the RMSE of the prediction result is 0.89609, which is smaller than the 0.91226 of the prediction result from Figure 5. Figure 13 is the error comparison of Figure 5 and Figure 12, the result shows the prediction error has decreased, and the overall prediction result after ELM error compensation has been improved. Although the error compensation value is very small, it can also further narrow the gap between the predicted value and the true value. Therefore, the overall prediction effect can be improved.

### 4.3. Comparison Studies of Error Compensation

**Figure 14:**
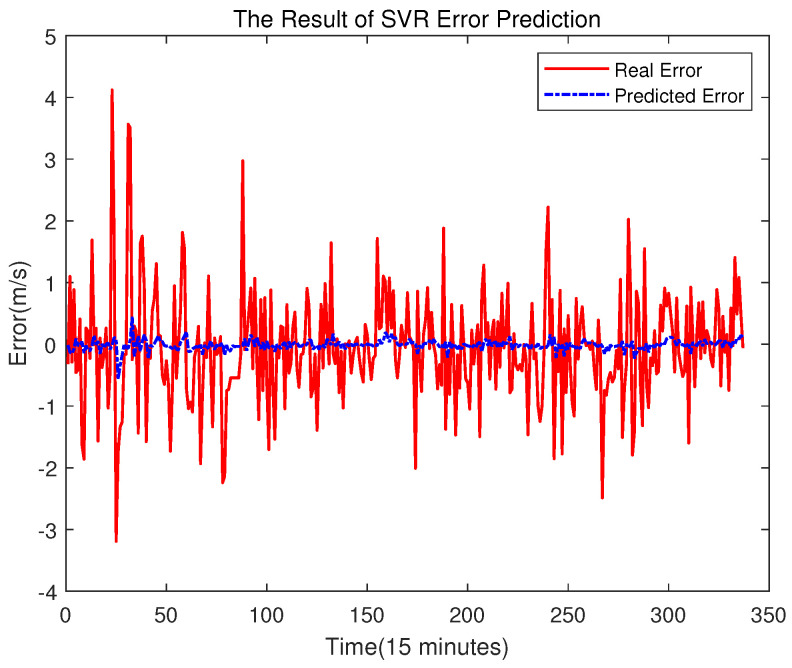
Error Prediction of SVR.

**Figure 15:**
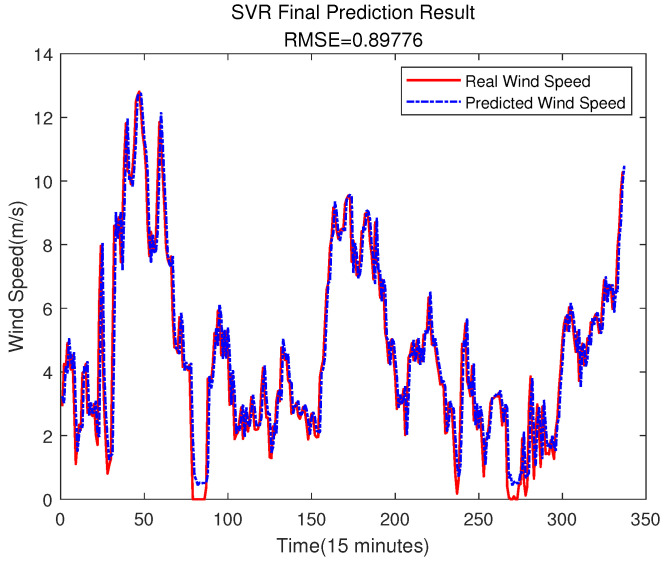
The Final Prediction Result of SVR.

**Figure 16:**
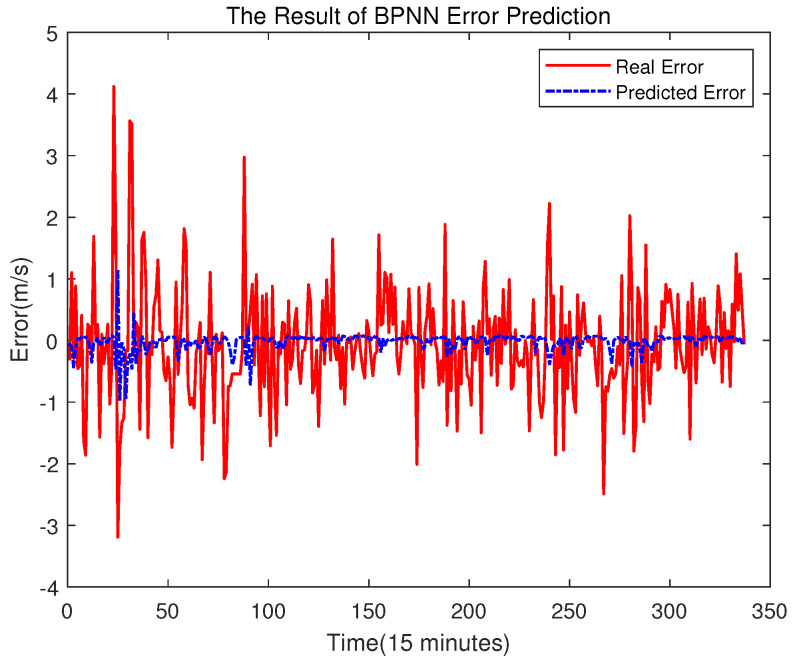
Error Prediction of BPNN.

**Figure 17:**
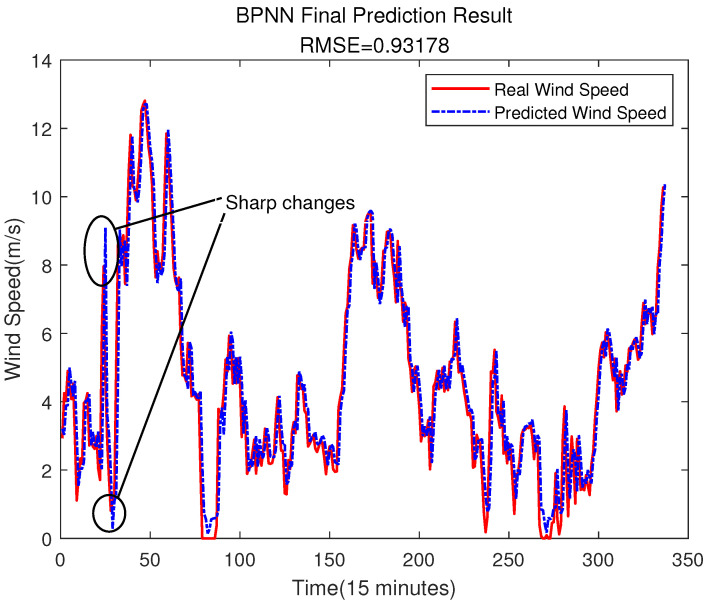
The Final Prediction Result of BPNN.

In order to prove the better effect of error prediction using ELM, this section compares the error prediction of SVR and BPNN. The training set of SVR and BPNN is the same as that of ELM. The results are shown in Figure 14, Figure 15, Figure 16 and Figure 17.

In this paper, the kernel function of SVR is RBF, σ is 0.0039, and the penalty factor is 3.8855. The neural nodes and hidden layers of the BPNN are 10 and 2, respectively.

It can be seen from the above figure that neither the SVR nor the BPNN method can predict the error very well. In Figure 15, the accuracy of the final prediction result after SVR error compensation is also improved compared to Figure 5, with the RMSE being 0.89776, but it is still slightly higher than the result of the ELM error compensation of 0.89609 in Figure 12. For the BPNN error compensation technique, there are always some sharp changes in the predicted wind speed, such as at time 25 and 29. This obviously will reduce the wind speed prediction accuracy.

**Figure 18:**
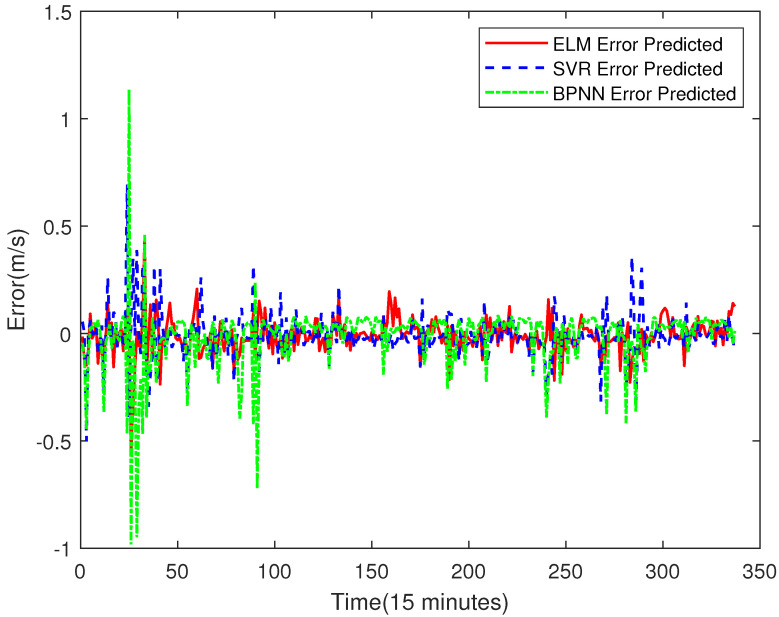
The Comparison of Error Prediction Results of ELM, SVR and BPNN.

**Figure 19:**
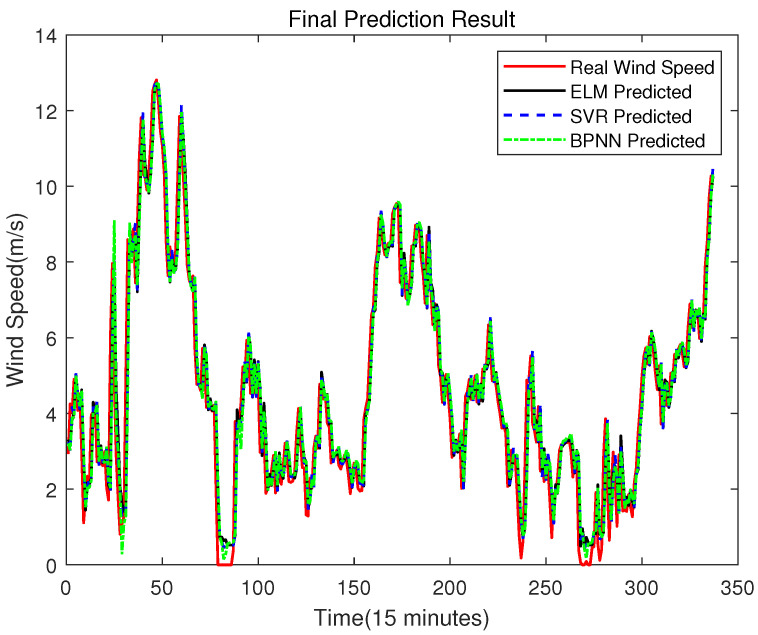
The Final Prediction Result of ELM, SVR and BPNN.

From Figure 18, the mean values of the error prediction results of ELM, SVR and BPNN models are −0.0108, −0.0112 and −0.0170, respectively, which indicates that the volatility of the BPNN prediction result is the highest, and the ELM prediction result is the smallest. In other words, the prediction result of the ELM error compensation is more accurate than that of the other two models. The final prediction result of ELM, SVR and BPNN after error compensation are shown in Figure 19.

Table 3 shows the evaluation indicators of prediction performance through ELM, SVR and BPNN error compensation, and whether RMSE, $R^{2}$ or ELM is better than the other two methods. Therefore, it is reasonable to use ELM for error compensation prediction.

## 5. Conclusions

In order to predict the univariate wind speed time series more accurately, this paper proposes a hybrid model based on ARMA-SVR to predict wind speed. ARMA is used to model the univariate series, and its PACF is obtained to guide the data division. Then, the divided data are used to train the SVR model to predict wind speed. This way can make full use of the original data without wasting machine performance, and it can improve the prediction effect. Then, in order to further improve the prediction accuracy, the error compensation method is adopted in this paper. Through a comparison of ELM, SVR and BPNN, it is proven that ELM is more suitable for error compensation prediction.

With the verification of real data, the ARMA-SVR-ELM hybrid model proposed in this paper can improve the accuracy of wind speed prediction compared to the direct forecasting method, and thus it can be applied in practice.

Meanwhile, there are still some areas worth improving in this study. For example, this study only uses historical wind speed time series to conduct the prediction task. How to use multi spatio-temporal scale features to achieve more accurate prediction results is a promising research direction.

## Figures and Tables

**Table 1:** The AIC Values of ARMA Model.

*p*	*q*	AIC	*p*	*q*	AIC	*p*	*q*	AIC	*p*	*q*	AIC
1	1	−0.939708317	3	5	−0.941061416	6	1	−0.931624951	8	5	−0.933236582
1	2	−0.938949696	3	6	−0.937277647	6	2	−0.930127525	8	6	−0.933216729
1	3	−0.937090829	3	7	−0.930057817	6	3	−0.938491989	8	7	−0.94568503
1	4	−0.935025071	3	8	−0.936440702	6	4	−0.933036538	8	8	−0.944814908
1	5	−0.933116954	4	1	−0.934974836	6	5	−0.938025992	9	1	−0.927770895
1	6	−0.931432686	4	2	−0.936073001	6	6	−0.936211633	9	2	−0.936251062
1	7	−0.929815103	4	3	−0.931061169	6	7	−0.935605297	9	3	−0.93489583
1	8	−0.936929991	4	4	−0.939470353	6	8	−0.931863081	9	4	−0.933116554
2	1	−0.938981997	4	5	−0.946239715	7	1	−0.929987002	9	5	−0.941774164
2	2	−0.936982456	4	6	−0.930322493	7	2	−0.931898552	9	6	−0.93679826
2	3	−0.934982158	4	7	−0.928406952	7	3	−0.926560847	9	7	−0.94487932
2	4	−0.933065546	4	8	−0.935086314	7	4	−0.927954063	9	8	−0.94366315
2	5	−0.931151868	5	1	−0.933156842	7	5	−0.937354724	10	1	−0.926252232
2	6	−0.940212875	5	2	−0.942303885	7	6	−0.933872907	10	2	−0.934431651
2	7	−0.931849886	5	3	−0.93629865	7	7	−0.94291323	10	3	−0.936059114
2	8	−0.930386906	5	4	−0.946208256	7	8	−0.941815759	10	4	−0.931216218
3	1	−0.936983426	5	5	−0.934978618	8	1	−0.928777357	10	5	−0.939906908
3	2	−0.934978248	5	6	−0.928315448	8	2	−0.929908894	10	6	−0.938448221
3	3	−0.935676116	5	7	−0.926457981	8	3	−0.936635588	10	7	−0.938985571
3	4	−0.933944129	5	8	−0.935428434	8	4	−0.937461583	10	8	−0.943233736

**Table 2:** The AIC Values of Error ARMA Model.

*p*	*q*	AIC	*p*	*q*	AIC	*p*	*q*	AIC	*p*	*q*	AIC
1	1	−0.917157036	3	5	−0.913248754	6	1	−0.915371665	8	5	−0.910804917
1	2	−0.918237417	3	6	−0.914624364	6	2	−0.911872347	8	6	−0.907870931
1	3	−0.917806459	3	7	−0.919656616	6	3	−0.915797823	8	7	−0.917843557
1	4	−0.919608918	3	8	−0.911261485	6	4	−0.914817412	8	8	−0.919087666
1	5	−0.917585799	4	1	−0.916886099	6	5	−0.912784914	9	1	−0.91017278
1	6	−0.915559577	4	2	−0.917403694	6	6	−0.908763361	9	2	−0.908661079
1	7	−0.911957637	4	3	−0.918582861	6	7	−0.920249027	9	3	−0.912515236
1	8	−0.911492457	4	4	−0.913309095	6	8	−0.917784858	9	4	−0.908129994
2	1	−0.919090626	4	5	−0.920639364	7	1	−0.914013316	9	5	−0.915859181
2	2	−0.917948222	4	6	−0.92012195	7	2	−0.912221398	9	6	−0.90542256
2	3	−0.916349042	4	7	−0.912692359	7	3	−0.913419954	9	7	−0.9146417
2	4	−0.914395162	4	8	−0.912323987	7	4	−0.911743622	9	8	−0.915471508
2	5	−0.915599578	5	1	−0.915574726	7	5	−0.910158108	10	1	−0.909411234
2	6	−0.917955537	5	2	−0.915642376	7	6	−0.918849939	10	2	−0.907795459
2	7	−0.910156087	5	3	−0.920895364	7	7	−0.916877493	10	3	−0.906402831
2	8	−0.913209174	5	4	−0.916511787	7	8	−0.918561658	10	4	−0.906187391
3	1	−0.918491847	5	5	−0.91495742	8	1	−0.912181595	10	5	−0.909218128
3	2	−0.919431816	5	6	−0.913918875	8	2	−0.910342013	10	6	−0.908371191
3	3	−0.914373558	5	7	−0.908787598	8	3	−0.914344249	10	7	−0.917539225
3	4	−0.915595298	5	8	−0.91541658	8	4	−0.910003375	10	8	−0.915065366

**Table 3:** The RMSE and $R^{2}$ of ELM, SVR and BPNN.

Evaluation Index	ELM	SVR	BPNN
RMSE	0.8961	0.8978	0.9318
$R^{2}$	0.9021	0.9017	0.8941

## Data Availability

The data is unavailable due to privacy security.

## Nomenclature

The following nomenclatures are used in this manuscript:

*X*	Wind speed time series
$X_{t}$	Current value of wind speed time series
$\alpha_{i}$	Autoregressive parameter
$\beta_{j}$	Moving average parameter
*p*	Partial Autocorrelation Coefficient
*q*	Autocorrelation Coefficient
$\epsilon_{t}$	White nose of time t
Φ(x)	RBF kernel function
ω	Coefficient vector of SVR model
*b*	Bias of SVR model
$x^{{}^{\prime }}$	Center of kernel function
*C*	Penalty factor of SVR
N	Number of Samples
$x_{i}$	Input data of SVR
$y_{i}$	Output targets of SVR
$\xi^{*}$, ξ	Slack variables of SVR
ε	Loss coefficient of SVR
*K*	Number of neurons of ELM
$\omega_{j}$	Random weights of ELM
$b_{j}$	Deviation of ELM
$\lambda_{j}$	Output Layer Weights of ELM
g(.)	Sigmoid activation function
$x_{i}$	The ith value of wind speed time series
${\hat{x}}_{i}$	The ith estimate value of wind speed time series
$\bar{x_{i}}$	Average value of samples
AC	Autocorrelation Functions
ACF	Autocorrelation Coefficient
ADF	Augmented Dickey-Fuller
AIC	Akaike Information Criterion
ARMA	Autoregressive Moving Average
BPNN	Backpropagation Neural Network
ELM	Extreme Learning Machine
PAC	Partial Autocorrelation Coefficient
PACF	Partial Autocorrelation Functions
RBF	Radical Basis Functions
SVR	Support Vector Regression
